# Regional Differential Decomposition and Formation Mechanism of Dynamic Carbon Emission Efficiency of China’s Logistics Industry

**DOI:** 10.3390/ijerph182413121

**Published:** 2021-12-12

**Authors:** Jingwen Yi, Yuchen Zhang, Kaicheng Liao

**Affiliations:** School of Economics and Management, Tongji University, Shanghai 200092, China; 2030483@tongji.edu.cn (J.Y.); zhang99111@tongji.edu.cn (Y.Z.)

**Keywords:** carbon emission, logistics industry, dynamic efficiency, DEA-Malmquist model, PVAR model

## Abstract

Among China’s five major industries, the logistics industry is the only one in which carbon emission intensity is continuing to increase, so it is of great importance in developing a low-carbon economy for China. Thus, some scholars have learned about carbon emission efficiency (CEE) in logistic industry recently; however, few of them have considered the inner structure, regional differentiation, or dynamic items of CEE. To fill this gap, we first calculate the dynamic carbon emission efficiency of China’s logistics industry (CEELI) (2001–2017) using the three-stage DEA-Malmquist model, and then using the Dagum Gini coefficient method, the Kernel Density Estimation (KDE), and the panel vector auto-regression (PVAR) model to analyze regional differential decomposition and their formation mechanism. The results indicate that the dynamic CEELI is ‘inefficient’ overall; it shows a decreasing trend, and the decline of dynamic efficiency mainly comes from technical backwardness rather than efficiency decline. Moreover, the domestic differences are gradually narrowing; the Gini inequality between regions and the density of trans-variation between regions are the main reasons for the gap between different regions and different periods.

## 1. Introduction

In the past few years, China has been developing economic globalization and international business rapidly, which has enabled it to become the second largest, economy as well as the largest contributor to carbon emissions, in the world. According to the statistics from the world bank in 2019, China is among the top three countries in the world in terms of carbon emissions, together with the European Union, and the USA. As a result, China has pledged to peak its carbon emissions by 2030 and reduce carbon emissions per unit of GDP by 40–45% from the 2005 level. [1] Judging from energy structure, the logistics industry has become one of the largest and most important sources of energy consumption and carbon emissions in China [2]. According to the China Energy Statistical Yearbook, the energy consumption of the logistics industry has increased from 114.47 million tons in 2000 to 436.17 million tons in 2018, with an average annual growth rate of 7.71%. Its share of energy consumption in China’s total energy consumption has risen from 7.79% to 9.24%, which has continued to increase in recent years. Under the enormous pressures of reducing carbon emissions and developing a low-carbon economy, the logistics industry plays a significant part in achieving these goals, given its high carbon emission levels of it [3].

As a bridge and link connecting production and consumption [4], the logistics industry occupies a special and unreplaceable position in the low-carbon economy for three reasons. First, the logistics industry consists of high energy consumption and low operational efficiency, making it a key industry for carbon emissions reduction [5]. Second, developing a low-carbon economy needs the support of the modern logistics industry, which will decrease the cost of low-carbon economy operations and carbon emissions reduction. Judging from extant relative studies of different industries’ carbon emissions, we know that among the five major industries (i.e., agriculture, industry, construction, commerce, and logistics), only the logistics industry’s carbon emission intensity shows a trend of continuous increase, while the other four industries have decreased by various degrees [6]. Third, with the advent of the internet economy and digital economy, the logistics industry not only bears the service pressure of the rapid development, but also produces a lot of carbon emissions [3]. In addition, taking into account the differences in economic development rates, institutions and geographical conditions in different regions, carbon emissions vary significantly, which makes it difficult to implement emission reduction policies in line with local conditions. Thus, while seeking to improve the CEELI, we must give priority to the actual differences in regional economic development levels to accurately grasp the real level in different regions and to guide the development of the regional low-carbon economy. It is of great academic value and practical importance to comprehensively evaluate the logistics industry’s CEE from the perspective of regional differentiation.

Based on this, we use the three-stage DEA-Malmquist model to measure dynamic CEELI from 2001 to 2017, and we use the Dagum Gini coefficient method to empirically test the sources of regional differentiation of CEELI at the provincial level. The PVAR model is chosen to explore the dynamic effects between efficiency change (*EC*) and technical change (*TC*), with the hope of empirically diagnosing the formation mechanism behind regional differences of CEELI and of providing a reference point for the decision making of enterprise regulators and relevant government departments. At the same time, this research can theoretically make up for the lack of research on dynamic CEE.

Compared with existing literature, we can summarize the contribution of this study as follows: (i) Regarding the research method, although many scholars have used the three-stage DEA model to study the static effect of CEE [7,8], we pay more attention to exploring CEE from a dynamic perspective. By introducing the *ML* index, we take CEE’s decomposition into consideration, which can help us to understand CEE’s internal structure more deeply and to give more reasonable and practical suggestions. (ii) Regarding the research content, most of the relative research has focused on CEE itself, for example, CEE’s evolution, regional differences, and spatial convergence. In contrast to this, we expand our research perspective to evaluate CEE’s internal structure and the formation mechanism of regional differences, based on the division of three major regions (according to the Seventh Five-Year Plan issued in 1986, China is divided into three major regions, namely, the Eastern (Hebei, Beijing, Shandong, Shanghai, Tianjin, Fujian, Jiangsu, Zhejiang, Guangdong, Hainan, and Liaoning), the Central (Jilin, Hubei, Jiangxi, Anhui, Shanxi, Hunan, Henan, Heilongjiang, Inner Mongolia), and the Western regions (Shaanxi, Guizhou, Chongqing, Guangxi, Sichuan, Gansu, Yunnan, Qinghai, Ningxia, Tibet, Xinjiang)). (iii) Regarding the research context, previous scholars have made contributions to industrial engineering [7], the construction industry [8], etc. Few of them have studied the CEE of China’s logistics industry. However, China’s prosperous development has brought the logistics industry to center stage, and this industry will play an essential and strategic role in the long run. We hope our research can offer some practical advice to public administration and reference experience to other developing countries, if possible.

The rest of this paper is arranged as follows: The second part is the literature review, combing through and reviewing the relevant research. The third part introduces the empirical test design of this study and rationalizes our research logic by introducing various research methods, measurement models, and data sources. The fourth part analyzes the temporal and spatial evolution of CEELI and decomposes the regional difference of it. The fifth part analyzes the internal structure of CEELI. The sixth part discusses our results. The final part provides some conclusions and policy implications.

## 2. Literature Review

Facing the goal of establishing high-quality economic growth, it is important for China to overcome environmental constraints and promote a low-carbon economy, for which improving CEE is an effective solution [9]. CEE originated from the concept of carbon productivity, which was first raised by Yamaji et al. in 1993. CEE refers to the amount of carbon dioxide required to produce each unit of economic output [10]. Up to now, the studies on CEE falls into three categories: (i) the calculation of CEE, (ii) the factors which affect CEE, and (iii) the analysis of regional differentiation of CEE. Most of these studies are concerned with evaluating CEE using different measurements.

Thanks to many scholars’ contributions, calculation methods have developed from single-factor measurement [11,12,13,14,15] to total-factor measurement [16,17] over the years. This study is also based on total-factor measurement. In the process of constructing the multi-input/multi-output system, this study examines the relationships among the multi-factor inputs/outputs such as labor, capital, and energy consumption.

Another hot area of research is CEE measurement for a region or industry. Scholars tend to choose two main models, the SFA (i.e., stochastic frontier analysis) model and the DEA (i.e., data environment analysis) model. The SFA model can study the effect of DMU efficiency difference and obtain more accurate results [18,19,20,21]. However, SFA has several obvious disadvantages: it needs a specific form of functions, and its assumptions must be satisfied strictly. Compared with SFA, DEA is more popular in scholars’ research, because the new objective enables the calculation of the weight of each index of input-output data estimation, avoiding errors caused by the parameters and subjectivity in the establishment of the model [8].

Although the DEA model has developed rapidly scholars have come to realize that traditional DEA is limited under certain conditions. Thus, the three-stage DEA model, a revised version of traditional DEA, was first raised by Fried et al. in 2002 [22]. By now, CEE measurements based on three-stage DEA are mainly designed statically, without considering dynamic performance changes. This limits the understanding of the dynamics of carbon emissions. Now, the main methods to explore CEE’s dynamics are the DEA-Malmquist index method and the DEA-Malmquist-Luenberger index method [16,23]. From the perspective of dynamic analysis, some scholars used the DEA-Malmquist index method to calculate China’s provincial total-factor energy efficiency and analyzed every region’s efficiency [24]. Additionally, some other scholars used this method to decompose China’s agricultural CEE [25]. However, in the study of CEE, there is little application of the DEA-Malmquist method, resulting in the existing dynamic research on carbon emission efficiency being slightly insufficient.

In conclusion, the research and exploration of these scholars undoubtedly laid an important theoretical and methodological foundation for the study of CEE in China. In this paper, we first measure the CEE of China’s logistics industry in 30 Chinese regions from 2001 to 2017 using the three-stage DEA-Malmquist model, then we analyze the regional differential decomposition and formation mechanism of CEE. (At present, there is no ‘logistics industry’ in the statistical industry classification system. And more than 80% of the logistics industry is from the transportation, warehousing, and postal industry, so we believe they can reflect the extensive development of the logistic industry. As such, we based have our study of the logistics industry on these industries.)

## 3. Methods and Data Sources

### 3.1. The Three-Stage DEA Model

We chose a three-stage DEA model to calculate the input/output of DMUs’ non-radial slacks. This model not only minimizes the effect of the external environment and random interference but can also obtain a more accurate measurement of dynamic CEELI. This model is composed of three stages:

#### 3.1.1. Stage One: The Traditional DEA Model

The traditional DEA can measure the effectiveness of DMU and the slack value of input/output. In CEE calculation, it is harder to control the output than the input, so for any DMU, the DEA-BCC model [26] can be expressed as:

(1) $$\begin{array}{c} \min\theta \\ st.\left\{ \begin{array}{l} \sum_{i=1}^{n}\lambda_{i}x_{ij}+s^{-}=\theta x_{0j}\\ \sum_{i=1}^{n}\lambda_{i}y_{ir}-s^{+}=y_{0r}\\ \sum_{i=1}^{n}\lambda_{i}=1\\ \begin{array}{ccc} \lambda_{i}\geq 0; & s^{+}\geq 0; & s^{-}\geq 0 \end{array} \end{array}\right. \end{array}$$

In this formula, *i* = 1, 2, …, *n*; *j* = 1, 2, …, *m*; *r* = 1, 2, …, *s*. *n* means the number of DMUs, *m* and *s* mean the number of input and output variables, respectively. *x_ij_* is the *j*th input factor of the *i*-decision unit, *y_ir_* is the *r*th output factor of the *i*-decision unit, and *θ* is the effective value of the decision unit.

#### 3.1.2. Stage Two: The SFA Model

The traditional DEA model is very sensitive to environmental variables and random errors and cannot objectively reflect the actual effectiveness of DMU management. Therefore, in the second stage, we chose the SFA model. In stage one, we explored the correlation between input slack and external environmental variables and random error terms and then adjusted input variables [27]. SFA can eliminate the influence of environmental and random factors by adjusting all DMUs to the same environment, finally reflecting the actual management level.

#### 3.1.3. Stage Three: The Adjusted DEA Model

The adjusted input data replaced the original one, and then efficiency was evaluated using the DEA-BCC model. In the end, the efficiency value of each DMU was obtained.

### 3.2. The Three-Stage DEA-Malmquist Model

We constructed the three-stage DEA-Malmquist model to analyze the dynamic changes of CEE. The specific model is described as follows: first, we used the traditional DEA-Malmquist model to analyze the changes in total factor productivity; second, we applied the SFA model to adjust input-output variables. Third, we put the adjusted input variables and original output variables into the DEA-Malmquist model for calculation.

In this model, the *ML* index (i.e., Malmquist index) is used to measure the change rate of total factor productivity [28]. According to the calculation method of the Malmquist index raised by Chung et al. [29], the *ML* index from period *t* to period *t* + 1 is:

(2) $$ML_{t}^{t+1}=\sqrt{\frac{\left[1+{\vec{D}}_{0}^{t}\left(x^{t},y^{t},b^{t};y^{t},-b^{t}\right)\right]}{\left[1+D_{0}^{t}\left(x^{t+1},y^{t+1},b^{t+1};y^{t+1},-b^{t+1}\right)\right]}•\frac{\left[1+{\vec{D}}_{0}^{t+1}\left(x^{t},y^{t},b^{t};y^{t},-b^{t}\right)\right]}{\left[1+D_{0}^{t+1}\left(x^{t+1},y^{t+1},b^{t+1};y^{t+1},-b^{t+1}\right)\right]}}$$

*ML* measures the change of productivity from period *t* to period *t* + 1, if *ML* < 1, CEE is decreased; if *ML* = 1, CEE is unchanged; if *ML* > 1, CEE is improved. The *ML* index concludes in two parts: one part measures efficiency change (*EC*), and the other part measures technical change (*TC*). The expression is as follows:

(3) $$ML_{t}^{t+1}=MLEFFCH_{t}^{t+1}•MLTECH_{t}^{t+1}$$

(4) $$MLEFFCH_{t}^{t+1}=\frac{1+{\vec{D}}_{0}^{t}(x^{t},y^{t},b^{t};y^{t},-b^{t})}{1+{\vec{D}}_{0}^{t+1}(x^{t+1},y^{t+1},b^{t+1};y^{t+1},-b^{t+1})}$$

(5) $$MLTECH_{t}^{t+1}=\sqrt{\frac{\left[1+{\vec{D}}_{0}^{t+1}\left(x^{t},y^{t},b^{t};y^{t},-b^{t}\right)\right]}{\left[1+D_{0}^{t}\left(x^{t},y^{t},b^{t};y^{t},-b^{t}\right)\right]}•\frac{\left[1+{\vec{D}}_{0}^{t+1}\left(x^{t+1},y^{t+1},b^{t+1};y^{t+1},-b^{t+1}\right)\right]}{\left[1+D_{0}^{t}\left(x^{t+1},y^{t+1},b^{t+1};y^{t+1},-b^{t+1}\right)\right]}}$$

*EC* measures the degree of approximation between each observed value and their respective production frontier. *TC* measures the change of production possibility boundary from stage *t* to stage *t* + 1. Among them, *EC* > 1 indicates efficiency change has increased. *EC* < 1 indicates efficiency change has decreased. *TC* > 1 indicates technical change has increased. *TC* < 1 indicates technical change has decreased. *ML* > 1 indicates CEE has increased. *ML* < 1 indicates CEE has declined.

### 3.3. Dagum Gini Coefficient Method

Based on the research method of Dagum [30], this paper divides the study area into eastern, central, and western economic regions, and uses the Dagum Gini coefficient to empirically test the regional differences and sources of CEE and the efficiency change (*EC*) and technical change (*TC*) of CEELI. The calculation formula of the Dagum Gini coefficient is as follows:

(6) $$G=\frac{\sum_{i=1}^{K}\sum_{j=1}^{K}\sum_{h=1}^{n_{i}}\sum_{r=1}^{n_{j}}\left|y_{ih}-y_{jr}\right|}{2n^{2}\mu }$$

In Formula (6), *G* represents the overall Gini coefficient. *K* represents the eastern, central, and western regions, *y_ih_* and *y_jr_* represent the real level of CEELI in any province and city in region *i* and region *j*, (*i* = 1, 2, …, *K*. *j* = 1, 2, …, *K*), respectively. *μ* is the average CEELI in provinces and cities, *n* is the number of provinces and cities, *n_i_* and *n_j_* are the number of provinces and cities in *i*(*j*) area, respectively. 

The Gini coefficient of Dagum [30] can be decomposed into three parts, namely, the Gini inequality within regions (*G_w_*), the net extended Gini inequality between regions (*G_rb_*), and the intensity of trans-variation between regions (*G_t_*). The relationship between them is *G* = *G_w_* + *G_rb_* + *G_t_*, where *G_w_* represents the distribution gap of CEELI in region *i*(*j*), *G_rb_* indicates the distribution gap of CEELI between *i*(*j*) regions, and *G_t_* represents the impact of the cross term of CEELI among regions on the overall Gini coefficient *G*. If *G_t_* = 0, the cross term of the energy carbon emission efficiency of logistics industry among regions does not exist. The Gini coefficient decomposition formula of Dagum (1997) is as follows:

(7) $$G_{ii}=\frac{\sum_{h=1}^{n_{i}}\sum_{r=1}^{n_{j}}\left|y_{ih}-y_{jr}\right|}{2n_{i}^{2}\mu_{i}}$$

(8) $$G_{w}=\sum_{i=1}^{K}\lambda_{i}s_{i}G_{ii}$$

(9) $$G_{ij}=\frac{\sum_{h=1}^{n_{i}}\sum_{r=1}^{n_{j}}\left|y_{ih}-y_{jr}\right|}{n_{i}n_{j}(\mu_{i}+\mu_{j})}$$

(10) $$G_{rb}=\sum_{i=2}^{K}\sum_{j=1}^{i-1}\left(\lambda_{j}s_{i}+\lambda_{i}s_{j}\right)G_{ij}D_{ij}$$

(11) $$G_{t}=\sum_{i=2}^{K}\sum_{j=1}^{i=1}\left(\lambda_{j}s_{i}+\lambda_{i}s_{j}\right)G_{ij}\left(1-D_{ij}\right)$$

In the above formula, (7) and (8) denote the Gini coefficient *G_ii_* and the contribution *G_w_* of the regional gap, (9) and (10) denote the Gini coefficient *G_ij_* and the contribution *G_rb_* of the regional gap. In addition, *λ_i_* = *n_i_*/*n*, *s_i_* = *λ_i_μ_i_/μ*, *i* = 1, 2, …, *K*. *D_ij_* = (*d_ij_* − *p_ij_*)/(*d_ij_* + *p_ij_*), indicating the relative economic impact of the *i* and *j* regions. *d_ij_* represents the total influence between regions *i* and *j*. When *μ_i_* > *μ_j_*, *d_ij_* is the weighted average of the carbon emission efficiency gap (*y_ih_* − *y_ir_*) of all logistics industries under the condition of *y_ih_ > y_ir_*. For the continuous density distribution functions *f_i_*(*y*) and *f_j_*(*y*), *d_ij_* can be expressed as:

(12) $$d_{ij}=\int_{0}^{\infty }\int_{0}^{y}\left(y-x\right)f_{j}(x)dxf_{j}(y)dy$$

*p_ij_* is the first-order moment of trans-variation, which can be understood as a weighted average of the energy carbon emission efficiency gap (*y_ih_* − *y_ir_*) of all logistics industries under the condition of *y_ih_* > *y_ir_*, when *μ_i_* > *μ_j_*, expressed as:

(13) $$p_{ij}=\int_{0}^{\infty }\int_{0}^{y}\left(y-x\right)f_{j}(x)dxf_{j}(y)dy$$

### 3.4. Data Resources

The panel data this research needed were collected from 30 provinces, municipalities, and autonomous regions in China from 2001 to 2017. (There are 34 provinces in China. Considering the lack of data, we excluded Tibet, Macau, Hong Kong, and Taiwan from the sample). The classification standard of ‘National Economic Industry Classification’ (The ‘Classification of National Economic Industries’ (GB/T4754-2003) divides China into six major industries. The six industries cover (1) agriculture, forestry, animal husbandry, and fishing; (2) the construction industry; (3) the transportation, warehousing, and telecommunications industry; (4) wholesale and retail; (5) accommodation and catering; (6) other industries. This paper focuses on the dynamic CEELI. The required data mainly came from the 2001–2017 *China Statistical Yearbook*, *China Energy Statistical Yearbook*, *China Science and Technology Statistical Yearbook*, *local statistical yearbooks*, and bulletins. A portion of the missing data was supplemented by research methods such as interpolation, exponential smoothing, and mean methods. To test the interval differences in the dynamic CEELI, the study is based on the three-main-region dividing criteria and makes an empirical analysis of the samples of different regions. Specific variables are selected as follows:

**Input-output variables**. In accordance with [7], we use *labor*, *capital stock*, and *total energy consumption* (i.e., the sum of the different energies consumed by the physical and immaterial sectors of production) as input variables. We divide the output variables into expected and unexpected outputs. The former corresponds to gross industrial product (i.e., industrial products or businesses that are sold or are for sale), while the latter meets the criteria for calculating carbon dioxide emissions from the National Inventory of greenhouse gases (IPCC, 2006). The following Formula (14) is used to calculate the carbon dioxide emissions from energy-burning in China’s provincial energy industry:

(14) $$CO_{2}=\sum_{i=1}^{3}E_{i}\times NCV_{i}\times CEC_{i}$$

In this formula, *E* is the energy consumption, *NCV* is the net calorific value of each fuel, and *CEC* is the carbon emission coefficient. After comparison, we chose the specific information in Table 1 for use in the Formula (14).

The specific information for the variables is shown in Table 2.

**Environmental variables:** When selecting environmental variables, the main criterion was that the variable has a significant impact on CEE but is also a factor that cannot be controlled by the DMU. Based on available data, variable indicators, and existing research [31,32], this study focused on economic energy, institutional environment, etc.; six indicators were selected as the environmental variables in this paper. They are *economic development level*, etc. The above indicators were measured with regional per capita GDP, etc., and detailed descriptions of the environment variables can be seen in Table 2.

## 4. Results

### 4.1. Overview of Dynamic CEELI

Table 3 reveals the evolution trend of the mean value of dynamic CEELI (*ML*) at the national level and in the eastern, the central, and the western regions from 2001 to 2017. To accurately analyze the dynamic CEELI, we also show the evolution trend of efficiency change (*EC*) and technical change (*TC*) of industrial carbon emission. Overall, the dynamic CEELI has decreased significantly from 2001 to 2017. Specifically, at the national level, the *ML* average value from 2001 to 2017 is 0.914, and the average annual growth rate is −0.027%, which means that the dynamic CEELI during this period is generally ‘inefficient’, and the efficiency has been gradually declining. From the perspective of dynamic efficiency decomposition, the average *EC* from 2001 to 2017 is 1.019, indicating that although technical efficiency was effective, there was an insignificant efficiency reversal. The average value of *TC* is 0.909, with an average annual growth rate of −0.026%, indicating that the technical change of CEELI is backward. From the above analysis, the loss of CEELI is mainly caused by technical regression.

Considering regional differences, the *ML* averages of the eastern, central, and western regions are 0.967, 0.891, and 0.877, respectively; the annual growth rates are −0.026%, −0.024%, and −0.031%, respectively. This indicates that the dynamic CEELI in China’s three major regions show a downward trend and an ‘inefficient’ state. In the spatial pattern, there is an obvious ‘east-central-west’ decreasing trend. From the interannual trend, the three regions all show negative growth. Among them, negative growth rates in the eastern (−0.026%) and central (−0.024%) regions are lower than the national average (−0.027%), while negative growth in the western (−0.031%) regions is more pronounced. The central region showed strong growth in *EC*, in contrast to the negative growth in the east (−0.004%) and west (−0.008%), reaching a growth of 0.007%. When it comes to *TC*, the negative growth rate of the central region ranks first among the three regions, for there is a big ‘central collapse’ dilemma in the central region; that is, it has been marginalized for a long time in policy, which the development of the western region makes more obvious, resulting in a small growth rate in the central region.

After analyzing the above phenomenon, we know that: First, the eastern region ranks first among three regions regarding CEE, because it has the advantages of location factors and early policy tilt, enabling the logistics development here to be more scientific. Second, although the western region has a policy tilt and a lot of potential investment opportunities, the regional infrastructure support and compatibility are poor, meaning that its resource advantages has not been released. Thus, the logistics development is lagging, and the CEE is the lowest among the three regions and shows obvious negative growth. Third, the central region has good natural conditions and abundant resources such as coal and oil, enabling logistics to begin to develop and take shape significantly. However, it also faces difficulties such as a low degree of opening-up, unreasonable energy structure, and extensive development, which explains why technical change is lagging.

### 4.2. Evolution of Dynamic CEELI

**Figure 1:**
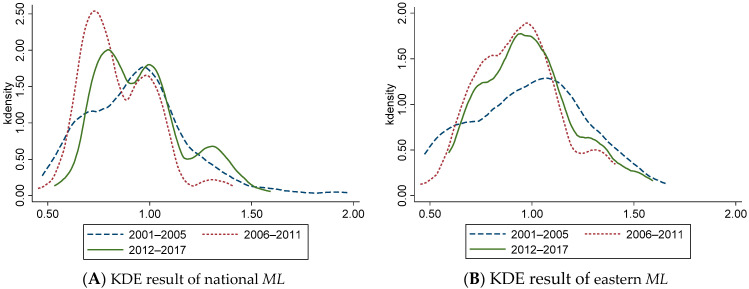

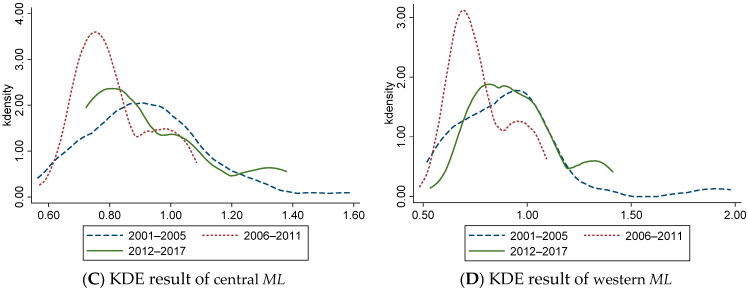
The Kernel Density Estimation Results of *ML* of China’s Logistics Industry. (**A**) KDE result of national *ML* (**B**) KDE result of eastern *ML* (**C**) KDE result of central *ML* (**D**) KDE result of western *ML*.

**Figure 2:**
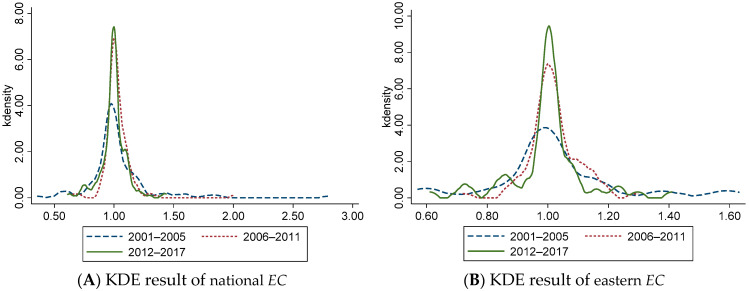

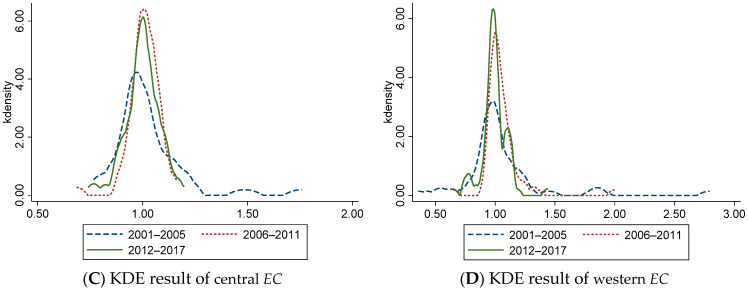
The Kernel Density Estimation Results of Efficiency Change of China’s Logistics Industry. (**A**) KDE result of national *EC* (**B**) KDE result of eastern *EC* (**C**) KDE result of central *EC* (**D**) KDE result of western *EC*.

**Figure 3:**
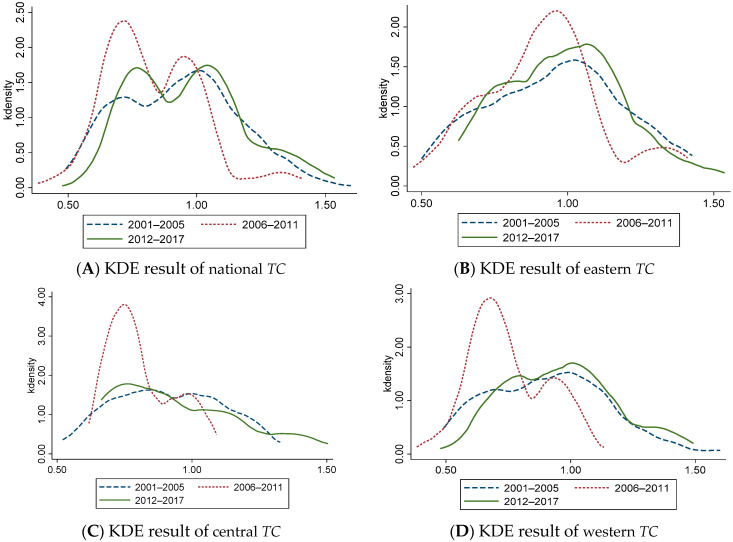
The Kernel Density Estimation Results of Technical Change of China’s Logistics Industry. (**A**) KDE result of national *TC* (**B**) KDE result of eastern *TC* (**C**) KDE result of central *TC* (**D**) KDE result of western *TC*.

To further understand the distribution and evolution of *ML*, *EC*, and *TC*, we divided the observation period into three stages, and selected 2001–2005, 2006–2011, and 2012–2017 for Kernel Density Estimation (KDE) (Figure 1, Figure 2 and Figure 3). It should be noted that KDE is basically the same as the information conveyed by the above general description, but there are some differences. The above overview describes the rules for changing data in time series, which is an intuitive presentation of data and cannot reflect more information. The function of KDE is to describe the distribution of sample data, which can reflect the convergence, polarization, and other related information. To a certain extent, KDE can provide empirical support for the above conclusions.

Figure 1A shows that from the national level, the *ML* shows a unimodal distribution from 2001 to 2005 and a bimodal distribution from 2006 to 2017, indicating that *ML* has a polarization trend from 2006 to 2017. The peak value of the main peak first increases and then decreases, and the width of the main peak first decreases and then increases, representing the absolute difference of *ML* in each region’s ‘narrowing-expanding’ trend during this period. From 2006 to 2017, the center of the curve shifted left obviously, indicating the overall decline of *ML*. Figure 1B shows that the center of the curve shifts left significantly from 2006 to 2017, representing a gradual decline in *ML* in the eastern region. Compared with 2001–2005, the main peak value increases significantly, and the width decreases during 2006–2017, indicating that the absolute difference of *ML* between provinces in the eastern region decreases. Figure 1C shows that the changing trend of *ML* in the central region is like that of the whole country. The overall *ML* shows a ‘decline-rise’ trend, and there is a polarization trend between 2006 and 2017. The absolute difference between provinces in the central region first decreases and then expands. The peak value decreases step by step, indicating that the polarization is alleviated and controlled. Figure 1D shows that the *ML* variation trend in the western region during the observation period is like that in the central region.

Figure 2A shows that, at the national level, *EC* shows a single peak distribution in each period from 2001 to 2017. The peak of the main peak is rising, and the width is gradually narrowing, indicating that the regional difference of *EC* is narrowing. Figure 2B shows that, from 2001 to 2011, *EC* has a unimodal distribution, and from 2012 to 2017, it is composed of one main peak and multiple side peaks, indicating that *EC* in the eastern region shows a trend of multi-polarization. The main peak gradually increases, and the width gradually narrows, indicating that the regional differences of *EC* among provinces in the eastern region gradually decreased during this period. Figure 2C shows that from 2001 to 2017, the peak value of the main peak continues to rise, and the width gradually narrows, indicating that the distance difference of *EC* between regions in the central region gradually decreases. At the same time, the center of the curve moves right, which represents the overall rise of *EC* in the central region from 2006 to 2017. Figure 2D shows that the *EC* curve has a unimodal distribution from 2001 to 2011, and there is the main peak and multiple side peaks from 2012 to 2017, representing the trend of multi-polarization of *EC* in the western region during this period. During this period, the peak value of the main peak gradually increases, and the width narrows, indicating that the regional difference of *EC* in the western region have gradually decreased during this period.

Figure 3A shows that from the national level, *TC* has always shown a stable bimodal distribution, indicating that the polarization trend is obvious. However, the peak value of the main peak decreases in a ladder form from 2012 to 2017, indicating that the polarization phenomenon has been improved and alleviated. The center of the curve moves left first and then right, indicating that *TC* shows a trend of ‘decrease- increase’. Figure 3B–D show that from 2001 to 2005 and from 2012 to 2017, the *TC* curve has a single peak distribution, while between 2006 to 2011 there appears a bimodal distribution, which indicates that the polarization trend of *TC* appears during this period, and the polarization phenomenon has been improved after 2012.

### 4.3. Decomposition of Regional Differences of Dynamic CEELI

The above analysis mainly describes the temporal and spatial variation of dynamic CEELI in various regions, but it does not describe regional differences or the sources of those differences. Therefore, we further explore the regional differences of dynamic CEELI.

**Figure 4:**
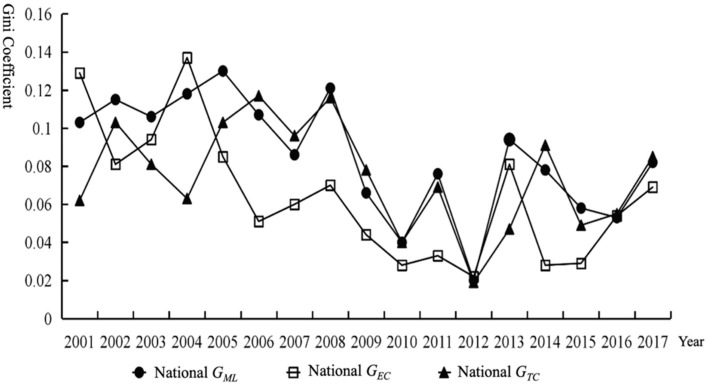
The Total Gini Coefficient of *ML*, *EC*, and *TC*.

Figure 4 shows the trend changes of the overall Gini coefficient of the dynamic efficiency (*ML*), efficiency change (*EC*), and technical change (*TC*) of dynamic CEELI from 2001 to 2017. From the changing trend of dynamic CEELI (*G_ML_*) in the logistics industry, *G_ML_* shows a fluctuating downward trend from 2001 to 2017. The Gini coefficient shows an average annual growth rate of −1.414%, indicating that the gap between *ML* levels of the logistics industry in China is gradually narrowing. From the perspective of interannual variation, the fluctuation of *G_ML_* reached the lowest point in the observation period (0.020) in 2012 and reached the highest point in the observation period (0.121) in 2008. From 2007 to 2016, the fluctuation of *G_ML_* is the most intense, and it experienced three peaks and valleys in eight years. From the changing trend of the efficiency change Gini coefficient (*G_EC_*) and technical change Gini coefficient (*G_TC_*) of industrial carbon emissions, the fluctuation trends of *G_EC_*, *G_TC_*, and *G_ML_* from 2001 to 2017 are similar: *G_EC_* decreases from 0.129 (2001) to 0.069 (2017). *G_TC_* shows a trend of ‘rising-decreasing-rising’ from 2001 to 2017, reaching a peak of 0.117 in 2006 and a minimum of 0.019 in 2012. Among them, *G_ML_*, *G_EC_*, and *G_TC_* reached their minimum at the same time in 2012.

According to the above analysis, the decrease in *G_EC_* is the main reason for the reduction in *G_ML_*, and the decrease in *G_TC_* also promotes the reduction in *G_ML_*, but this effect varies at different time stages. Before 2007, the changing trend of *G_EC_* and *G_TC_* was always the opposite. During this period, the change of *G_EC_* came earlier than that of *G_ML_* 1–2 years, and the change of *G_TC_* was later than that of *G_ML_* 1–2 years. Therefore, it can be proven that, during this period, the influence of *G_EC_* on G_ML_ was greater than that of *G_TC_* on *G_ML_*. After 2007, the changes of the three tend to be synchronized, and the negative growth rate of *G_TC_* was greater than that of *G_EC_*, so the impact of *G_TC_* on *G_ML_* was greater than that of *G_EC_* on *G_ML_*.

According to Table 4, from 2001 to 2017, *G_ML_* in the eastern region showed an upward trend, rising from 0.072 (2001) to 0.106 (2017), the average annual growth rate of which is 2.45%. *G_ML_* in the central region decreased from 0.082 in 2001 to 0.028 in 2017, reaching an average annual growth rate of −6.50%. *G_ML_* in the western region decreased from 0.134 (2001) to 0.073 (2017), and its average annual growth rate is −3.72%. This shows that, from the horizontal comparison, *ML* has the largest intra-group gap in the eastern region. In terms of *G_EC_*, from 2001 to 2017, the region with the largest decline in *G_EC_* is the western region, the average annual growth rate of which reaches −6.00%. This indicates that, from the horizontal comparison, the largest gap within the group of *EC* was in the western region. In terms of *G_TC_*, the evolution trend in these three regions shows a great difference from 2001 to 2017. Specifically, the eastern and western regions show an upward trend, while the central region shows a downward trend.

The above analysis shows that: First, the intra-group gap within the central region is the smallest. This perhaps originates from the fact that, in 2006, the State Council issued relevant opinions to build the central region into an important comprehensive transportation hub, which caused the central region to form a relatively unified development strategy. Since 2007, the overall level of the logistics industry in the central region has been improved, so the regional differences have gradually narrowed. Second, the intra-group difference in the eastern region is the largest, which may be due to the great inconsistency in the level of logistics development. The modernization of the eastern and southern coastal areas started earlier, with a relatively specialized, networked, and intensive logistics industry. The logistics links such as transportation, warehousing, and distribution are well connected, so the carbon emission efficiency is high. However, although the northeast region has rich resources (i.e., coal, oil, and natural gas), there exist some problems such as the excessive development of resources and the upstream of the industrial chain, for which the CEE here is backward. Third, the *EC* in the western region has a large intra-group difference because the economic development mode here is extensive, and the environmental cost is high. Although the logistics industry has begun to develop in recent years against the background of ‘Western Development’, there is still a problem of uneconomic scale, and the difference in technical efficiency is widening.

Table 5 indicates that the interregional differences of *ML*, *EC*, and *TC* are at different levels and show great differences in the evolution trend. In terms of the mean value, the differences of *ML*, *EC*, and *TC* between the eastern and western regions rank first. From the perspective of interannual variation, the differences of *ML*, *EC*, and *TC* between the central and western regions maintain a negative growth rate, indicating that the central and western regions are shrinking year by year. It can also be seen that the increase in *ML* difference between the eastern and central regions is mainly due to *TC*, while the decrease in *ML* difference between the eastern and western regions is mainly due to *EC*.

The above analysis shows that: First, the huge differences between the eastern and western regions should not be explained by differences in economic development level, infrastructure construction, and energy structure design. Instead, we should consider the low level of convergence and the difficulty of cluster effect construction caused by the large geographical distance between the two regions. Second, the gap between the central and western regions is narrowing year by year, which may be due to the strong technological catch-up in the western regions against the background of ‘Western Development’. Geographic proximity leads to cooperation and clustering, which has significantly reduced the technical efficiency difference between the central and western regions.

### 4.4. The Source Decomposition and Contribution Rate of Dynamic CEELI

Table 6 reports the sources and contributions of *ML*, *EC*, and *TC* differences. Table 5 shows that, from the perspective of *ML*, the contribution rates of the Gini inequality within regions (*G_w_*), the net extended Gini inequality between regions (*G_rb_*), and the intensity of trans-variation between regions (*G_t_*) from 2001 to 2017 are 31.17%, 37.73%, and 32.71%, respectively. The source of the *ML* gap in China is *G_rb_*, *G_t_*, and *G_w_*. From the perspective of interannual change, *G_w_* maintains a negative growth rate close to 0 during the observation period. The fluctuation of *G_rb_* is relatively large, showing growth in general. Similarly, *G_t_* has also seen big swings, with overall growth negative but faster in recent years. What should be noticed is that the contribution rate of *G_t_* has gradually increased in recent years, indicating that the impact of cross-overlap between different regions on the overall difference is gradually increasing. *G_t_* is mainly used to identify the overlapping phenomenon between regions. For example, *ML* in the eastern region is significantly higher than that in the western region, but the efficiency value of some provinces with lower *ML* levels in the eastern region may be lower than that of provinces with higher *ML* level in the western region. This also means that in recent years, there are a small number of cities with high *ML* development in the eastern, central, and western region. *ML* shows ‘discrete’ distribution in space, and there is no agglomeration phenomenon.

From the perspective of *EC*, the average contribution rates of *G_w_*, *G_rb_*, and *G_t_* from 2001 to 2017 are 31.36%, 27.31%, and 41.33%, respectively. The source of the gap between different regions in China is *G_t_*, *G_w_*, and *G_rb_*, and *G_t_* is the main cause of the gap. From the perspective of inter-annual changes, *G_w_* is like that of *G_w_* in *ML* samples, with little fluctuation and negative growth of nearly zero. The changes of *G_rb_* and *G_t_* are reversed. The former has a large negative growth rate, while the latter has a large growth rate, which indicates that the inter-group gap of *EC* has less and less influence on the overall difference, and the overlapping between different regions has more and more influence on the overall difference.

From the perspective of *TC*, the average contribution rates of *G_w_*, *G_rb_*, and *G_t_* from 2001 to 2017 are 29.47%, 40.78%, and 29.75%, respectively. The source of the *TC* gap in China is *G_rb_*, *G_t_*, and *G_w_*, and *G_rb_* is the main cause of the gap. From the perspective of inter-annual changes, *G_w_* is like that of *G_w_* in *ML* samples, with little fluctuation and negative growth of nearly zero. The changes of *G_rb_* and *G_t_* are reversed. The former has a large growth rate, while the latter has a large negative growth rate, which indicates that the inter-group gap of *TC* has an increasing impact on the overall difference, and the overlapping between different regions has an increasingly smaller impact on the overall difference.

## 5. Additional Analysis

### 5.1. Stationarity Test of Variables

The above analysis can partly explain the static relationship between *ML* on the one hand and *EC* and *TC* on the other, but it cannot explain the dynamic relationship between *ML* and *EC* and *TC* or the relationship between *EC* and *TC*. This is because *EC* and *TC* are generated by the decomposition of the *ML* index, and there is a certain internal relationship between them. The VAR model has the advantage of allowing each component to be an endogenous variable. At the same time, the intertemporal length of our data is 17 years, which also meets the requirements of time series samples. Therefore, we use the PVAR model to test the dynamic relationship between the three. Before the PVAR model analysis, it is necessary to test the stability of each variable and determine the optimal lag order of the model. Results are shown in Table 7. For the stationarity test, this paper selected *LLC*, *IPS*, *ADF*, and *PP* test statistics to determine whether each variable belongs to the stationary sequence. We found that the variables of each region sample are stationary sequences, so the original data could be directly modeled.

### 5.2. Granger Causality Test

The Granger causality test can accurately determine the correlation between variables. However, before the test, we needed to determine the optimal delay order of the model. According to the existing research methods, we used the order with the largest number of test values as the final optimal lag order of the model. The test results are shown in Table 8: The optimal lag order is 4 in the samples of the whole country and the western region, and 3 in the samples of the eastern and central regions. Moreover, the Granger causality test is quite different for different samples. At the national level, all the original assumptions were rejected, indicating that there was a significant interaction between *EC*, *TC*, and *ML*. The samples in the eastern region show that *ML* is affected by *EC* and *TC*, respectively, but not by the combined effect of the two. *EC* is not affected by *ML*, *TC*, or their interaction. On the contrary, *TC* is affected by *ML*, *EC*, and their interaction. The central region’s results are like the national results; however, because the optimal lag order is 3, the causal relationship is more sensitive. The samples in the western region show that only *EC* is the cause of *ML* change, and only *EC* is the cause of *TC* change, while the change of *EC* is caused by the joint action of *ML*, *TC*, and the interaction between them.

### 5.3. PVAR Model Analysis of the Dynamic CEELI

In this part, we continue to sample the three PVAR model system OLS estimation. As seen in Table 9, the test shows that: From the national level, in the early development of *ML*, the impact of *EC* and *TC* are negative and significant, while the impact of *ML* on *EC* is negative in the long term, and the impact of *ML* on *TC* is a long-term positive. The development of *EC* has long been positively influenced by *TC*, but its influence on *TC* is negative.

From the perspective of the eastern region, *ML* has a self-enhancement mechanism in the short term, but both *EC* and *TC* inhibit the increase in *ML*, and *ML* also significantly promotes *TC*. It is worth noting that *ML*, *EC*, and *TC* have no significant effect on *EC*; the reason may be that the technical change of the eastern region has become saturated, and the pulling effect is not obvious.

From the perspective of the central region, *ML* has a self-enhancement mechanism in the short term and is positively affected by *EC* and *TC* in the long term. The growth of *ML* has a lagging negative impact on the development of *EC*, and the growth of *EC* has a lagging positive impact on *TC*.

From the perspective of the western region, *ML* is negatively affected by both *EC* and *TC*, and its growth has a short-promoting effect on the development of *EC*. The growth of *EC* mainly comes from the self-promotion of the lag phase 3 and the positive impact of *TC*.

### 5.4. Impulse Analysis and Variance Decomposition of the Dynamic CEELI

**Figure 5:**
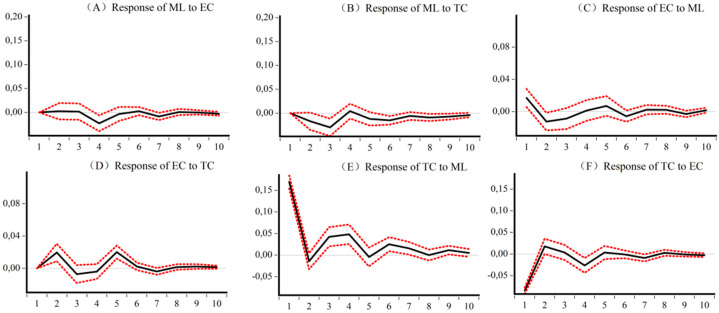
Impulse response results of *ML*, *EC*, and *TC* of National CEE. (**A**) Response of *ML* to *EC* (**B**) Response of *ML* to *TC* (**C**) Response of *EC* to *ML* (**D**) Response of *EC* to *TC* (**E**) Response of *TC* to *ML* (**F**) Response of *TC* to *EC*.

**Figure 6:**
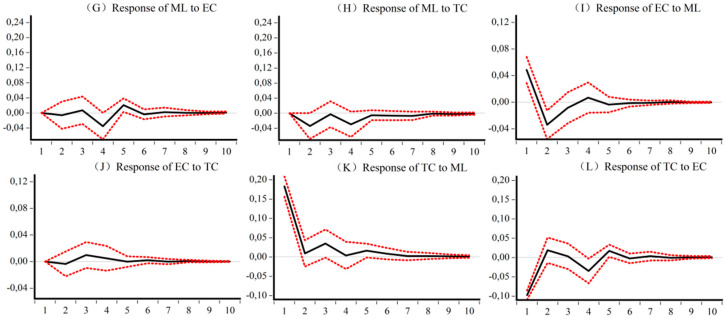
Impulse response results of *ML*, *EC*, and *TC* of Eastern CEE. (**G**) Response of *ML* to *EC* (**H**) Response of *ML* to *TC* (**I**) Response of *EC* to *ML* (**J**) Response of *EC* to *TC* (**K**) Response of *TC* to *ML* (**L**) Response of *TC* to *EC*.

**Figure 7:**
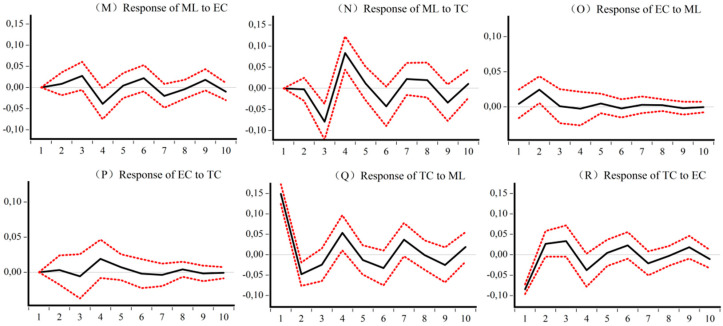
Impulse response results of *ML*, *EC*, and *TC* of Central CEE. (**M**) Response of *ML* to *EC* (**N**) Response of *ML* to *TC* (**O**) Response of *EC* to *ML* (**P**) Response of *EC* to *TC* (**Q**) Response of *TC* to *ML* (**R**) Response of *TC* to *EC*.

**Figure 8:**
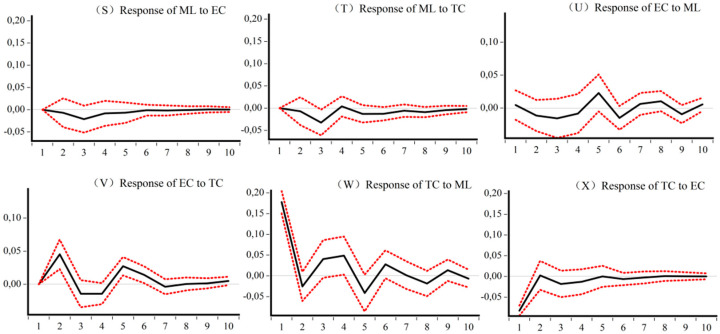
Impulse response results of *ML*, *EC*, and *TC* of Western CEE. (**S**) Response of *ML* to *EC* (**T**) Response of *ML* to *TC* (**U**) Response of *EC* to *ML* (**V**) Response of *EC* to *TC* (**W**) Response of *TC* to *ML* (**X**) Response of *TC* to *EC*.

Figure 5, Figure 6, Figure 7 and Figure 8 are the impulse response results among *ML*, *EC*, and *TC* of four different samples. The abscissa is the response period of the impact, set to 10 periods. Order is the degree of influence of variables. The median curve represents the impulse response function, and the curves on either side represent quantile estimates of 95% and 5%, respectively.

Figure 5 shows, for the national sample, first that there are significant differences in the impact of *EC* and *TC* on *ML* across the country, as *TC* is the driving force for the initial growth of *ML*, while *EC* plays a catalytic role in the medium term. Second, *ML* improves the development of *EC* and *TC*, but it has a greater impact on *TC*. Third, the national *TC* is the driving force for the growth of *EC*, but *EC* has a greater inhibitory effect on *TC* in the early stages.

Figure 6 shows, for the eastern sample, first that *EC* and *TC* have a positive impact on *ML*, but the effect time varies. In the early stage, *TC* plays a role, and in the middle stage, *EC* and *TC* play a role together. Second, *ML* in the eastern region has an inhibitory effect on *EC* and *TC*, but the effect on *EC* is greater than that on *TC*. Third, *EC* in the eastern region delays the development of *TC* in the early stage, while *TC* has a promoting effect on the development of *EC* in the middle stage.

Figure 7 shows, for the central sample, first that both *EC* and *TC* also contribute to the development of *ML* and are more intense than the national sample. Second, the development of *ML* in the central region will have a weak positive effect on *EC* in the early stage, but a strong negative impact on *TC*. Third, *TC* in the central region is the source of promoting *EC* growth in the medium term, while *EC* shows a strong inhibitory effect on *TC* in the early stage.

Figure 8 shows, for the western sample, first that *EC* and *TC* have a positive effect on *ML* in the early stage, but the impact time is relatively short. Second, the development of *ML* in the western region has an initial negative effect on *EC* and *TC*, but the difference is that *ML* has a greater impact on *TC* than *EC*. Third, *EC* promotes *TC* until the shock disappears, while the effect of *TC* on *EC* is unstable.

Table 10 conveys the following useful information. First, the contribution rates of *ML* and *EC* to *TC* are greater than the impact of *TC* itself, indicating that the growth of *TC* is mainly driven by external factors. Second, in the impact of *EC* and *TC* on *ML*, the contribution of *TC* is greater than that of *EC*. Third, in the impact of *ML* and *TC* on *EC*, the contribution of *ML* is greater than that of *TC*, but both are less than the contribution of *EC* itself.

## 6. Discussion

In contrast to the existing literature on CEE [7,8], we have shed light on China’s logistics industry regarding its essential role in developing a low-carbon economy. This study represents a noteworthy innovation. The results of the three-stage DEA-Malmquist model show that the total dynamic CEELI is ‘inefficient’ during our research period, and great differences exist among the three main regions. Considering *ML*, the eastern ranks first because of advantages such as location and policy tilt, and the western is at the bottom because of imperfect infrastructure and resources [5]. When it comes to *TC*, the negative growth rate of the central region ranks first among the three regions because of its big ‘central collapse’ dilemma. Using the Dagum Gini coefficient method, we know that the gap between the *ML* levels of the logistics industry in China is gradually narrowing, and in 2012, all three kinds of gap (i.e., *G_ML_*, *G_EC_*, *G_TC_*) reached their lowest value. This phenomenon can be explained by two reasons: (1) Driven by the Internet economy, residents’ consumption has broken through the limitations of geographical location, and online shopping has become normal. The logistics industry in the central and western regions has also developed rapidly, narrowing the gap with the eastern regions. (2) Since 2012, new-energy vehicles have been promoted, bringing much cleaner transportation to the logistics industry. This has also narrowed the regional differences. Through decomposing the intra-group and inter-group difference, we find that net extended Gini inequality between regions (*G_rb_*) is the main cause of total regional difference. What is more, the results of the PVAR model reveal the correlation among *ML*, *EC*, and *TC*.

Regarding research methods, we also have designed a more stable model. Firstly, a three-stage DEA-Malmquist model is introduced to measure dynamic CEELI from 2001 to 2017. Secondly, the Dagum Gini coefficient method is adapted to empirically test the sources of regional differentiation of CEELI at the provincial level. Thirdly, the PVAR model is chosen to test the dynamic effects between efficiency change (*EC*) and technical change (*TC*), to diagnose the formation mechanism of the regional differences of CEELI.

Additionally, we give the following possible explanations for the ‘inefficient’ status of CEELI. (1) The mode of transport is not properly configured. There are many shortcomings in logistic infrastructure in the western region [4], and the connection level between regions is not high, which accounts for the fact that the development of the logistics industry has not yet exerted a cluster effect. (2) The energy structure is not reasonable. For now, the western region has a single energy structure. Although it has rich energy reserves, such as coal, wind, and solar energy reserves, the development and utilization of new energy are seriously inadequate, with backward technology and equipment and low efficiency [20]. Therefore, the CEELI in the western region is the lowest of all three. (3) The regional linkage is not sufficient. A big gap exists in the level of development among different regions which can be explained by the lack of regional cooperation to some extent. Without a coordinated and organic logistic system, the distribution would be larger, and lots of resources would be wasted unnecessarily.

## 7. Conclusions and Policy Implications

### 7.1. Conclusions

This study analyzes the differential regional decomposition and formation mechanism of dynamic CEELI from 2001 to 2017. Using the three-stage DEA-Malmquist model, Dagum Gini coefficient method, and Kernel Density Estimation, we draw the following conclusions.

First, the dynamic CEELI is ‘inefficient’ overall, and it shows a decreasing trend. Both efficiency change and technology change have declined, but the decline of the latter is greater, indicating that the decline of CEELI mainly comes from technological backwardness. In the samples from different regions, the dynamic CEELI generally shows a downward trend, and all of them are ‘inefficient’. In terms of spatial pattern, it shows a gradually decreasing trend of ‘east-central-west’. The efficiency change of the central region rises obviously, and the technology change lags obviously.

Second, in terms of regional differentiation, the overall Gini coefficients of China’s logistic industrial carbon emission dynamic efficiency, efficiency change, and technology change all show a decreasing tendency, indicating that domestic differences are gradually narrowing. In turn, the narrowing of the efficiency change gap explains the narrowing of the dynamic efficiency gap. Considering the contribution rate of performance, we find that the Gini inequality between regions and the density of trans-variation between regions are the main reasons for the gap between different regions and periods.

Third, in the analysis of the internal structure of dynamic efficiency, we find that there is an interaction between efficiency change, technology change, and dynamic efficiency. However, in terms of the intensity, direction, and persistence of the interaction, significant differences exist between regions. For example, at the national level, dynamic efficiency is negatively affected by efficiency change and technology change in the early stage. Efficiency change has long been positively affected by technology change and negatively affected by dynamic efficiency. On the other hand, technology change has long been positively affected by dynamic efficiency and negatively affected by efficiency change.

### 7.2. Policy Implications

Based on our findings and actual situation, we propose some policy suggestions:

First, the key to improving dynamic CEELI is advancing efficiency and technology at the same time. Since technical change is the main reason for the backward CEELI, we must try to solve the problem of backward technology. Combined with the rapid development of the logistics industry, more and more modern transportation vehicles are being designed. Thus, it is necessary to introduce advanced equipment to the central region: this could include constructing intelligent logistic systems and developing the logistic information platform in order to improve the level of production technology and reduce energy consumption.

Second, we are supposed to fully consider regional heterogeneity and develop a logistics industry development policy. For example, in the western region, they should make good use of their own conditions to develop wind, solar energy, and other new energy, and improve the utilization of new energy. In addition, with the development of digitalization, it is necessary to build an intelligent transportation system to improve the operational efficiency of the logistics industry. While accelerating the upgrading of industrial structure, they should encourage the introduction and development of the new energy industry. Moreover, it is very important to motivate the coordinated development of the eastern and the western regions.

Third, breaking regional barriers in the logistics industry and strengthening regional communication are of great importance. Considering the imbalanced development of CEELI, the balanced development of regional CEELI can be achieved by reducing the gap between efficiency change and technical change in various regions. We should promote technology exchange and cooperation regarding regional energy utilization, and in particular, accelerate the technological diffusion of eastern coastal cities to the Central and Western backward areas. Additionally, we should build a harmony for collaboration to realize regional linkage.

## Figures and Tables

**Table 1:** Fuel, *NCV*, and *CEF*.

Fuel	Coal	Coke	Crude Oil	Gasoline	Kerosene	Diesel Oil	FUEL OIL	Natural Gas
*NCV* [kj/kg(m^3^)]	20,908	283,435	41,816	43,070	43,070	46,252	41,816	38,931
*CEF* [kg·CO_2_/TJ]	95,333	107,000	73,300	70,000	71,500	74,100	77,400	56,100

Notes: The data for *NCV* is from China Energy Statistical Yearbook, and the data for *CEF* is from IPCC 2006.

**Table 2:** Indicators for Measuring and Calculating the Dynamic CEELI.

Index	Variable	Index Composition	Unit
Input	Labor Input	Number of employments at the end of the year by region in logistics industry	(10,000 people)
	Capital Input	Capital stock ^1^	(RMB 100 million yuan)
	Total Energy Consumption	The total energy consumption of each province over the years	(10,000 tons)
Output	Gross Logistic Production	The gross logistic production value of each region is regarded as the expected output	(RMB 100 million yuan)
	Carbon Dioxide Emission	CO_2_ emissions in various regions as undesired output	(10,000 tons)
Environment	The Level of Economic Development	GDP per capita	
	Industrial Structure	The ratio of logistic production to GDP ^2^	(%)
	Energy Structure	The ratio of agricultural carbon emissions to total energy consumption.	(%)
	The Level of Technological Innovation	The ratio of government investment in environmental governance to GDP	(%)
	Government Environmental Regulation	The ratio of R&D expenditure to GDP	(%)
	Degree of Opening-up	The ratio of total import and export to GDP	(%)

^1^ Calculation method: First, based on the construction method of [33] of capital stock, the price deflator of the investment data of various industries in this article was calculated using [34]’s deflation index construction method. Then, drawing on the provincial depreciation rate and base period capital stock data calculated using the [35] method, according to the perpetual inventory method, the provincial capital stock data of the three industries in this paper were obtained. Finally, through the calculation of the amount of capital in the base period and the selection of depreciation rates and current investment indicators, the total investment in fixed assets of the whole society was then deflated. ^2^ Calculation method: According to the calculation formula in the ‘Guidelines for National Greenhouse Gas Inventories’ compiled by the IPCC, 2006.

**Table 3:** The Dynamic CEELI.

Regional	Dynamic Efficiency (*ML*)				Efficiency Change (*EC*)				Technical Change (*TC*)			
	National	Eastern	Central	Western	National	Eastern	Central	Western	National	Eastern	Central	Western
2001	1.222	1.253	1.163	1.241	1.100	1.080	0.985	1.225	1.143	1.176	1.175	1.078
2002	0.950	1.065	0.875	0.891	0.960	1.013	0.985	0.879	1.009	1.056	0.889	1.064
2003	0.930	0.910	1.020	0.873	0.956	0.968	0.973	0.928	0.993	0.956	1.046	0.985
2004	0.712	0.633	0.763	0.751	1.120	0.959	1.188	1.236	0.646	0.671	0.655	0.611
2005	0.897	1.068	0.791	0.805	1.029	1.098	0.981	0.996	0.873	0.986	0.806	0.809
2006	0.797	0.896	0.812	0.673	1.015	0.979	0.987	1.080	0.802	0.923	0.822	0.652
2007	0.966	1.078	0.878	0.921	1.026	0.992	0.993	1.094	0.961	1.088	0.886	0.888
2008	0.818	0.981	0.750	0.699	1.061	1.078	1.023	1.077	0.776	0.916	0.734	0.661
2009	0.706	0.742	0.711	0.663	1.019	1.016	1.009	1.031	0.698	0.733	0.711	0.646
2010	1.024	1.074	1.004	0.988	1.028	1.024	1.014	1.046	0.998	1.051	0.991	0.947
2011	0.771	0.828	0.750	0.728	1.032	1.032	1.036	1.027	0.748	0.801	0.724	0.710
2012	1.041	1.053	1.037	1.030	0.991	0.979	1.004	0.991	1.051	1.077	1.034	1.040
2013	0.956	0.942	0.951	0.977	0.908	0.891	0.913	0.923	1.052	1.049	1.044	1.062
2014	0.852	0.921	0.787	0.835	1.015	1.025	1.022	0.999	0.843	0.900	0.770	0.846
2015	0.820	0.875	0.776	0.799	1.003	1.038	0.992	0.974	0.818	0.844	0.783	0.822
2016	1.296	1.300	1.299	1.289	1.004	1.046	0.963	0.995	1.297	1.250	1.356	1.297
2017	0.788	0.826	0.786	0.748	1.060	1.009	1.098	1.083	0.750	0.824	0.717	0.699
Mean	0.914	0.967	0.891	0.877	1.019	1.013	1.010	1.034	0.909	0.959	0.891	0.872
Annual Growth Rate	−0.027	−0.026	−0.024	−0.031	−0.002	−0.004	0.007	−0.008	−0.026	−0.022	−0.030	−0.027

**Table 4:** Intra-group Differences in *ML*, *EC*, and *TC* of CEELI.

Year	*G_ML_*			*G_EC_*			*G_TC_*		
	Eastern	Central	Western	Eastern	Central	Western	Eastern	Central	Western
2001	0.072	0.082	0.134	0.087	0.055	0.202	0.068	0.03	0.072
2002	0.101	0.05	0.132	0.069	0.021	0.129	0.086	0.049	0.123
2003	0.141	0.066	0.074	0.133	0.025	0.091	0.101	0.049	0.065
2004	0.121	0.085	0.097	0.132	0.125	0.094	0.062	0.051	0.058
2005	0.107	0.086	0.112	0.079	0.067	0.09	0.108	0.052	0.076
2006	0.113	0.069	0.049	0.049	0.025	0.059	0.112	0.055	0.037
2007	0.087	0.057	0.061	0.036	0.026	0.103	0.079	0.062	0.095
2008	0.123	0.054	0.066	0.055	0.042	0.097	0.126	0.035	0.064
2009	0.09	0.037	0.026	0.03	0.057	0.041	0.104	0.043	0.048
2010	0.055	0.009	0.035	0.027	0.018	0.034	0.055	0.009	0.034
2011	0.105	0.029	0.048	0.037	0.03	0.027	0.1	0.014	0.048
2012	0.031	0.013	0.01	0.02	0.024	0.019	0.028	0.011	0.009
2013	0.141	0.037	0.071	0.103	0.044	0.079	0.064	0.036	0.031
2014	0.118	0.013	0.053	0.015	0.011	0.051	0.124	0.021	0.08
2015	0.068	0.018	0.047	0.03	0.018	0.028	0.068	0.012	0.038
2016	0.07	0.029	0.046	0.064	0.052	0.033	0.064	0.033	0.048
2017	0.106	0.028	0.073	0.078	0.03	0.075	0.1	0.027	0.075
Mean	0.097	0.045	0.067	0.061	0.039	0.074	0.085	0.035	0.059
Annual Growth Rate	2.45%	−6.50%	−3.72%	−0.68%	−3.72%	−6.00%	2.44%	−0.66%	0.26%

**Table 5:** Inter-group differences in *ML*, *EC*, and *TC* of CEELI.

Year	*G_ML_*			*G_EC_*			*G_TC_*		
	Eastern-Central	Eastern-Western	Central-Western	Eastern-Central	Eastern-Western	Central-Western	Eastern-Central	Eastern-Western	Central-Western
2001	0.081	0.108	0.113	0.077	0.151	0.149	0.053	0.073	0.056
2002	0.101	0.126	0.100	0.053	0.103	0.081	0.089	0.107	0.104
2003	0.115	0.113	0.082	0.092	0.118	0.064	0.086	0.088	0.062
2004	0.122	0.123	0.094	0.147	0.137	0.111	0.057	0.067	0.060
2005	0.129	0.133	0.102	0.080	0.090	0.081	0.108	0.112	0.067
2006	0.101	0.119	0.078	0.040	0.059	0.049	0.100	0.134	0.081
2007	0.094	0.087	0.061	0.032	0.072	0.071	0.092	0.100	0.082
2008	0.123	0.138	0.063	0.051	0.077	0.077	0.115	0.139	0.056
2009	0.071	0.074	0.041	0.044	0.036	0.050	0.081	0.089	0.053
2010	0.039	0.050	0.025	0.023	0.031	0.028	0.038	0.051	0.026
2011	0.082	0.092	0.042	0.035	0.033	0.029	0.074	0.087	0.035
2012	0.024	0.022	0.012	0.023	0.020	0.022	0.023	0.021	0.010
2013	0.103	0.112	0.059	0.080	0.094	0.065	0.053	0.051	0.034
2014	0.087	0.094	0.041	0.013	0.094	0.035	0.093	0.106	0.061
2015	0.061	0.065	0.036	0.028	0.033	0.024	0.052	0.056	0.031
2016	0.055	0.061	0.039	0.063	0.052	0.043	0.057	0.060	0.043
2017	0.082	0.099	0.054	0.063	0.081	0.056	0.084	0.101	0.054
Mean	0.086	0.095	0.061	0.055	0.075	0.061	0.074	0.085	0.054
Annual Growth Rate	0.14%	−0.57%	−4.49%	−1.17%	−3.81%	−5.94%	2.96%	2.03%	−0.20%

**Table 6:** The Results of Source Decomposition and Contribution Rate.

	Dynamic Efficiency (*ML*)						Efficiency Change (*EC*)						Technical Change (*TC*)					
Year	Contribution Value			Rate (%)			Contribution Value			Rate (%)			Contribution Value			Rate (%)		
	*G_w_*	*G_rb_*	*G_t_*	*G_w_*	*G_rb_*	*G_t_*	*G_w_*	*G_rb_*	*G_t_*	*G_w_*	*G_rb_*	*G_t_*	*G_w_*	*G_rb_*	*G_t_*	*G_w_*	*G_rb_*	*G_t_*
2001	0.032	0.016	0.055	31.22	15.40	53.39	0.041	0.047	0.041	31.60	36.70	31.70	0.02	0.019	0.023	31.82	30.87	37.31
2002	0.033	0.046	0.035	28.91	40.19	30.90	0.025	0.031	0.025	30.65	38.80	30.56	0.03	0.036	0.036	29.42	35.31	35.28
2003	0.033	0.034	0.04	30.81	31.67	37.53	0.03	0.01	0.053	32.12	11.12	56.77	0.025	0.02	0.036	30.89	24.28	44.84
2004	0.034	0.042	0.041	28.99	35.78	35.23	0.039	0.057	0.041	28.36	41.65	29.99	0.019	0.021	0.023	30.62	33.16	36.21
2005	0.035	0.071	0.024	26.90	54.81	18.29	0.027	0.026	0.033	31.37	30.42	38.21	0.028	0.048	0.027	27.64	46.55	25.81
2006	0.028	0.063	0.016	26.00	58.71	15.29	0.016	0.022	0.013	30.52	43.43	26.05	0.026	0.076	0.015	21.97	65.11	12.92
2007	0.024	0.047	0.015	28.18	54.84	16.98	0.019	0.022	0.019	32.09	36.89	31.01	0.027	0.049	0.021	27.92	50.57	21.51
2008	0.031	0.08	0.011	25.23	65.73	9.04	0.022	0.011	0.037	31.62	15.65	52.73	0.029	0.011	0.075	25.12	65.15	9.72
2009	0.019	0.025	0.022	28.48	38.58	32.93	0.014	0.005	0.025	31.40	11.10	57.50	0.024	0.028	0.026	30.26	35.90	33.84
2010	0.012	0.019	0.008	31.06	48.55	20.40	0.009	0.007	0.012	32.36	23.94	43.70	0.012	0.024	0.004	30.28	59.08	10.65
2011	0.023	0.03	0.024	29.74	38.98	31.28	0.011	0.002	0.02	32.54	5.68	61.78	0.021	0.028	0.021	29.96	40.39	29.66
2012	0.006	0.005	0.008	32.74	25.69	41.57	0.007	0.005	0.009	32.12	25.33	42.55	0.006	0.009	0.003	31.25	50.25	18.50
2013	0.03	0.008	0.056	59.25	8.77	59.25	0.027	0.008	0.047	32.55	10.13	57.33	0.015	0.004	0.028	32.56	7.94	59.50
2014	0.024	0.035	0.019	30.88	45.17	23.95	0.009	0.006	0.014	30.69	20.50	48.81	0.029	0.034	0.028	31.48	37.43	31.09
2015	0.016	0.027	0.014	28.38	47.38	24.24	0.009	0.015	0.006	30.39	50.64	18.97	0.015	0.016	0.018	30.29	33.54	36.17
2016	0.017	0.002	0.034	32.56	3.73	63.71	0.017	0.019	0.018	31.91	34.63	33.46	0.017	0.017	0.021	30.23	32.45	37.31
2017	0.025	0.023	0.035	30.55	27.42	42.02	0.021	0.019	0.029	30.79	27.67	41.54	0.025	0.038	0.022	29.23	45.27	25.50
Mean	0.025	0.034	0.027	31.17	37.73	32.71	0.020	0.018	0.026	31.36	27.31	41.33	0.022	0.028	0.025	29.47	40.78	29.75
Annual Growth Rate	−1.53%	2.29%	−2.79%	−0.13%	3.67%	−1.49%	−4.10%	−5.50%	−2.14%	−0.16%	−1.75%	1.70%	1.40%	4.43%	−0.28%	−0.53%	2.42%	−2.35%

**Table 7:** Results of Stationarity Test.

Region	Index	Mode (C, T, L)	*LLC*	*IPS*	*ADF*	*PP*	Result	Region	Index	Mode (C, T, L)	*LLC*	*IPS*	*ADF*	*PP*	Result
National	*ML*	(C, 0, 0)	−5.796 ***	−8.660 ***	183.896 ***	556.844 ***	Stationary	Central	*ML*	(C,0,0)	−4.030 ***	−4.731 ***	54.906 ***	212.911 ***	Stationary
			[0.000]	[0.000]	[0.000]	[0.000]					[0.000]	[0.000]	[0.000]	[0.000]	
	*EC*	(C, 0, 0)	−5.828 ***	−8.405 ***	179.731 ***	484.511 ***	Stationary		*EC*	(C,0,0)	−4.401 ***	−4.396 ***	51.065 ***	102.570 ***	Stationary
			[0.000]	[0.000]	[0.000]	[0.000]					[0.000]	[0.000]	[0.000]	[0.000]	
	*TC*	(C, 0, 0)	−7.657 ***	−8.888 ***	189.724 ***	498.923 ***	Stationary		*TC*	(C,0,0)	−4.627 ***	−4.791 ***	55.246 ***	175.758 ***	Stationary
			[0.000]	[0.000]	[0.000]	[0.000]					[0.000]	[0.000]	[0.000]	[0.000]	
Eastern	*ML*	(C, 0, 0)	−3.930 ***	−5.954 ***	75.291 ***	202.347 ***	Stationary	Western	*ML*	(C,0,0)	−2.229 ***	−4.268 ***	53.699 ***	141.586 ***	Stationary
			[0.000]	[0.000]	[0.000]	[0.000]					[0.0129]	[0.0000]	[0.000]	[0.000]	
	*EC*	(C, 0, 0)	−3.127 ***	−5.551 ***	71.532 ***	188.586 ***	Stationary		*EC*	(C,0,0)	−2.683 ***	−4.566 ***	57.134 ***	193.355 ***	Stationary
			[0.001]	[0.000]	[0.000]	[0.000]					[0.004]	[0.000]	[0.000]	[0.000]	
	*TC*	(C, 0, 0)	−6.527 ***	−6.607 ***	83.958 ***	213.243 ***	Stationary		*TC*	(C,0,0)	−2.185 ***	−3.920 ***	50.520 ***	109.922 ***	Stationary
			[0.000]	[0.000]	[0.000]	[0.000]					[0.014]	[0.000]	[0.000]	[0.000]	

Note: “*”, “**”, and “***” all indicate passing the test at the significance level of 10%, 5%, and 1%; the *p*-value is in square brackets; the empirical results retain three decimal places.

**Table 8:** Results of Granger Causality Test.

Null Hypothesis	National		Eastern		Central		Western	
	Ratio	Result	Ratio	Result	Ratio	Result	Ratio	Result
The change in *EC* is not the cause of the change in *ML* (df_A)	21.746 *** [0.000]	YES	7.645 * [0.054]	YES	24.65 *** [0.000]	YES	11.427 * [0.022]	YES
The change in *TC* is not the cause of the change in *ML* (df_A)	17.950 *** [0.001]	YES	6.273 * [0.099]	YES	30.182 *** [0.000]	YES	5.803 [0.214]	NO
The changes in *EC* and *TC* are not the cause of changes in *ML* simultaneously (df_B)	29.568 *** [0.000]	YES	10.556 [0.103]	NO	33.158 *** [0.000]	YES	12.218 [0.142]	NO
The changes in *ML* are not the cause of changes in *EC* (df_A)	35.241 *** [0.000]	YES	0.842 [0.840]	NO	6.583 * [0.087]	YES	32.847 *** [0.000]	YES
The change in *TC* is not the cause of the change in *EC* (df_A)	38.605 *** [0.000]	YES	0.759 [0.859]	NO	6.722 * [0.081]	YES	38.413 *** [0.000]	YES
The changes in *ML* and *TC* are not the cause of changes in *EC* simultaneously (df_B)	45.693 *** [0.000]	YES	6.808 [0.339]	NO	12.435 * [0.053]	YES	43.689 *** [0.000]	YES
The changes in *ML* are not the cause of changes in *TC* (df_A)	26.514 *** [0.000]	YES	7.629 * [0.054]	YES	13.806 *** [0.003]	YES	6.236 [0.182]	NO
The change in *EC* is not the cause of the change in *TC* (df_A)	17.560 *** [0.002]	YES	6.913 * [0.075]	YES	12.839 ** [0.005]	YES	9.128 * [0.058]	YES
The changes in *ML* and *EC* are not the cause of changes in *TC* simultaneously (df_B)	32.357 *** [0.000]	YES	16.113 ** [0.013]	YES	13.84978 0.0314	NO	12.817 [0.118]	NO
N	390		154		126		130	
df_A	4		3		3		4	
df_B	8		6		6		8	

Note: “*”, “**” and “***” all indicate that the test passed the test at the significance level of 10%, 5%, and 1%; the *p*-value in brackets; YES and NO respectively indicate whether the test passed or not. The empirical results retain three decimal places.

**Table 9:** Results of OLS.

**Index**	**National**			**Index**	**Western**		
	***ML***	***EC***	***TC***		***ML***	***EC***	***TC***
*ML*(−1)	0.359	−0.626 ***	0.621 **	*ML*(−1)	−0.012	−1.244 ***	0.497
	(0.267)	(0.159)	(0.272)		(0.417)	(0.285)	(0.446)
*ML*(−2)	1.029 ***	0.197	0.979 ***	*ML*(−2)	0.885 *	0.581 *	0.674
	(0.283)	(0.168)	(0.289)		(0.463)	(0.316)	(0.494)
*ML*(−3)	0.108	0.080	−0.045	*ML*(−3)	−0.127	0.022	−0.245
	(0.235)	(0.139)	(0.239)		(0.360)	(0.246)	(0.384)
*ML*(−4)	0.053	−0.411 ***	0.157	*ML*(−4)	0.239	−0.409 **	0.265
	(0.191)	(0.113)	(0.195)		(0.237)	(0.162)	(0.253)
*EC*(−1)	−0.372 *	0.285 **	−0.406 *	*EC*(−1)	−0.177	0.734 ***	−0.403
	(0.209)	(0.124)	(0.212)		(0.299)	(0.204)	(0.319)
*EC*(−2)	−0.649 ***	−0.262 **	−0.539 **	*EC*(−2)	−0.682 **	−0.548 **	−0.439
	(0.215)	(0.127)	(0.218)		(0.315)	(0.216)	(0.337)
*EC*(−3)	−0.026	−0.055	0.076	*EC*(−3)	0.181	−0.054	0.284
	(0.188)	(0.112)	(0.192)		(0.261)	(0.179)	(0.279)
*EC*(−4)	−0.117	0.292 ***	−0.186	*EC*(−4)	−0.274	0.275 **	−0.293
	(0.161)	(0.096)	(0.164)		(0.203)	(0.138)	(0.216)
*TC*(−1)	−0.513 *	0.596 ***	−0.736 ***	*TC*(−1)	−0.189	1.223 ***	−0.655
	(0.272)	(0.162)	(0.277)		(0.425)	(0.290)	(0.453)
*TC*(−2)	−0.876 ***	−0.266	−0.787 ***	*TC*(−2)	−0.786 *	−0.719 **	−0.486
	(0.289)	(0.172)	(0.295)		(0.464)	(0.317)	(0.495)
*TC*(−3)	0.229	-0.083	0.393	*TC*(−3)	0.394	−0.094	0.596
	(0.241)	(0.143)	(0.245)		(0.363)	(0.248)	(0.388)
*TC*(−4)	0.0004	0.488 ***	−0.164	*TC*(−4)	−0.325	0.519 ***	−0.435
	(0.2000)	(0.119)	(0.204)		(0.256)	(0.175)	(0.273)
C	1.740 ***	0.774 ***	1.601 ***	C	1.776 ***	0.710 **	1.555 ***
	(0.306)	(0.181)	(0.312)		(0.402)	(0.275)	(0.429)
**Index**	**Eastern**			**Index**	**Central**		
	***ML***	***EC***	***TC***		***ML***	***EC***	***TC***
*ML*(−1)	1.158 *	0.042	1.053 *	*ML*(−1)	−0.122	0.058	−0.239
	(0.625)	(0.343)	(0.567)		(0.511)	(0.387)	(0.589)
*ML*(−2)	0.258	−0.292	0.500	*ML*(−2)	2.684 ***	0.263023	2.618 ***
	(0.637)	(0.350)	(0.579)		(0.731)	(0.554)	(0.843)
*ML*(−3)	1.058 *	−0.14158	1.064 *	*ML*(−3)	−2.415 ***	−1.125 **	−1.679 **
	(0.607)	(0.333)	(0.551)		(0.579)	(0.439)	(0.668)
*EC*(−1)	−1.112 **	−0.397	−0.736	*EC*(−1)	0.007	−0.149	0.171
	(0.570)	(0.313)	(0.517)		(0.384)	(0.291)	(0.443)
*EC*(−2)	−0.110	0.114	−0.238	*EC*(−2)	−1.854 ***	−0.262	−1.756 ***
	(0.579)	(0.318)	(0.525)		(0.516)	(0.391)	(0.595)
*EC*(−3)	−1.113 **	0.099	−1.135 **	*EC*(−3)	1.706 ***	0.727 **	1.240 **
	(0.562)	(0.309)	(0.510)		(0.438)	(0.332)	(0.505)
*TC*(−1)	−1.271 **	−0.132	−1.077 *	*TC*(−1)	−0.089986	0.108995	−0.084878
	(0.639)	(0.350)	(0.580)		−0.49484	−0.37497	−0.57081
*TC*(−2)	−0.143	0.228	−0.340	*TC*(−2)	−2.866 ***	−0.181	−2.865 ***
	(0.651)	(0.357)	(0.591)		(0.730)	(0.553)	(0.842)
*TC*(−3)	−0.954	0.167	−0.989 *	*TC*(−3)	2.615 ***	1.165 **	1.891 ***
	(0.617)	(0.338)	(0.560)		(0.596)	(0.452)	(0.688)
C	3.192 ***	1.319 **	2.858 ***	C	1.172	0.445	1.516 *
	(1.092)	(0.599)	(0.991)		(0.738)	(0.559)	(0.851)

Note: “*”, “**”, and “***” all indicate that they passed the test at the significance level of 10%, 5%, and 1%; the standard errors are in parentheses.

**Table 10:** Results of Variance Decomposition.

**Period**	**National**											
	**Variance Decomposition of *ML***				**Variance Decomposition of *EC***				**Variance Decomposition of TC**			
	**S.E.**	***ML***	***EC***	***TC***	**S.E.**	***ML***	***EC***	***TC***	**S.E.**	***ML***	***EC***	***TC***
1	0.189	100.000	0.000	0.000	0.112	2.319	97.681	0.000	0.192	77.570	19.485	2.945
2	0.191	99.202	0.015	0.783	0.116	3.296	93.825	2.879	0.195	75.864	19.727	4.408
3	0.197	96.951	0.020	3.028	0.117	3.808	92.973	3.220	0.202	75.580	18.540	5.880
4	0.205	95.855	1.283	2.862	0.117	3.816	92.856	3.329	0.209	75.522	18.838	5.641
5	0.205	95.488	1.305	3.207	0.119	4.013	89.902	6.084	0.210	74.867	18.688	6.445
6	0.207	95.008	1.299	3.693	0.119	4.220	89.683	6.098	0.212	74.886	18.343	6.771
7	0.208	94.804	1.454	3.741	0.119	4.253	89.559	6.188	0.213	74.861	18.392	6.748
8	0.208	94.616	1.452	3.931	0.120	4.285	89.518	6.197	0.213	74.681	18.363	6.956
9	0.208	94.513	1.448	4.040	0.120	4.337	89.435	6.227	0.214	74.670	18.291	7.039
10	0.208	94.466	1.464	4.070	0.120	4.356	89.404	6.240	0.214	74.643	18.289	7.068
**Period**	**Eastern**											
	**Variance Decomposition of *ML***				**Variance Decomposition of *EC***				**Variance Decomposition of TC**			
	**S.E.**	***ML***	***EC***	***TC***	**S.E.**	***ML***	***EC***	***TC***	**S.E.**	***ML***	***EC***	***TC***
1	0.230	100.000	0.000	0.000	0.126	14.850	85.150	0.000	0.209	76.695	21.682	1.623
2	0.233	97.836	0.067	2.096	0.135	19.308	80.624	0.068	0.212	74.717	21.875	3.408
3	0.235	97.774	0.155	2.071	0.136	19.522	79.884	0.593	0.215	75.166	21.255	3.579
4	0.240	94.227	2.289	3.484	0.136	19.692	79.591	0.717	0.220	71.841	22.810	5.348
5	0.241	93.500	3.007	3.493	0.136	19.731	79.553	0.716	0.221	71.513	23.154	5.333
6	0.242	93.421	3.025	3.555	0.136	19.733	79.527	0.740	0.222	71.447	23.095	5.458
7	0.242	93.335	3.030	3.635	0.136	19.732	79.528	0.740	0.222	71.366	23.094	5.540
8	0.242	93.334	3.030	3.636	0.136	19.731	79.527	0.743	0.222	71.367	23.090	5.543
9	0.242	93.329	3.030	3.642	0.136	19.731	79.527	0.743	0.222	71.362	23.088	5.550
10	0.242	93.326	3.031	3.643	0.136	19.731	79.526	0.743	0.222	71.360	23.088	5.551
**Period**	**Central**											
	**Variance Decomposition of *ML***				**Variance Decomposition of *EC***				**Variance Decomposition of TC**			
	**S.E.**	***ML***	***EC***	***TC***	**S.E.**	***ML***	***EC***	***TC***	**S.E.**	***ML***	***EC***	***TC***
1	0.150	100.000	0.000	0.000	0.114	0.138	99.862	0.000	0.173	73.701	23.744	2.555
2	0.154	99.674	0.299	0.026	0.119	4.294	95.642	0.064	0.182	73.926	23.735	2.339
3	0.177	77.351	2.659	19.989	0.120	4.272	95.424	0.304	0.202	61.263	21.934	16.803
4	0.204	62.766	5.568	31.666	0.121	4.205	93.043	2.751	0.224	55.445	20.656	23.899
5	0.205	62.617	5.581	31.803	0.122	4.335	92.589	3.076	0.225	55.503	20.588	23.909
6	0.213	60.286	6.228	33.486	0.122	4.368	92.529	3.102	0.233	53.759	20.167	26.074
7	0.218	60.273	6.780	32.947	0.122	4.414	92.388	3.197	0.238	53.742	20.061	26.197
8	0.219	59.773	6.758	33.469	0.122	4.442	92.253	3.305	0.239	53.420	19.958	26.622
9	0.224	58.509	7.126	34.365	0.122	4.466	92.211	3.323	0.244	52.527	19.785	27.687
10	0.225	58.513	7.245	34.242	0.122	4.467	92.205	3.328	0.245	52.588	19.777	27.635
**Period**	**Western**											
	**Variance Decomposition of *ML***				**Variance Decomposition of *EC***				**Variance Decomposition of TC**			
	**S.E.**	***ML***	***EC***	***TC***	**S.E.**	***ML***	***EC***	***TC***	**S.E.**	***ML***	***EC***	***TC***
1	0.187	100.000	0.000	0.000	0.128	0.125	99.875	0.000	0.199	79.454	17.117	3.429
2	0.191	99.730	0.136	0.134	0.136	0.824	88.159	11.016	0.203	78.632	16.616	4.751
3	0.197	95.868	1.296	2.835	0.138	2.098	86.082	11.820	0.209	77.818	16.407	5.776
4	0.201	95.810	1.419	2.771	0.139	2.441	84.859	12.700	0.215	78.324	15.790	5.886
5	0.203	95.373	1.504	3.123	0.144	4.807	79.737	15.456	0.220	78.326	15.080	6.595
6	0.204	95.026	1.491	3.483	0.145	5.759	78.200	16.041	0.223	78.185	14.835	6.979
7	0.204	94.945	1.497	3.558	0.146	5.925	78.008	16.068	0.223	78.170	14.844	6.986
8	0.204	94.775	1.490	3.735	0.146	6.390	77.635	15.975	0.224	78.160	14.713	7.128
9	0.205	94.731	1.489	3.780	0.146	6.780	77.304	15.916	0.224	78.219	14.655	7.127
10	0.205	94.720	1.489	3.791	0.147	6.903	77.104	15.993	0.224	78.222	14.639	7.140

## Data Availability

Data available in a publicly accessible repository. The data presented in this study are openly available in https://www.cnki.net, 8 December 2021.
